# *Salmonella* Type III Secretion System Effectors

**DOI:** 10.3390/ijms26062611

**Published:** 2025-03-14

**Authors:** Micah J. Worley

**Affiliations:** Department of Biology, University of Louisville, Louisville, KY 40292, USA; micah.worley@louisville.edu; Tel.: +1-502-852-0966

**Keywords:** *Salmonella*, *Salmonella* pathogenicity island-1, *Salmonella* pathogenicity island-2, type III effectors

## Abstract

*Salmonella* is estimated to infect between 200 million and over 1 billion people per year. The exact number is not known, as many cases go unreported. Integral to the pathogenesis of *Salmonella*, as well as numerous other Gram-negative pathogens, is its type III effectors. *Salmonella* possesses two distinct type III secretion systems, encoded by *Salmonella* pathogenicity island-1 and *Salmonella* pathogenicity island-2. Together, they secrete at least 49 type III effectors into host cells that are collectively responsible for many of the virulence attributes of this pathogen. These virulence factors facilitate the invasion of host cells, induce and attenuate inflammation, and change the migratory properties of infected phagocytes, among other things. The effects of all type III effectors on *Salmonella* virulence are discussed.

## 1. Introduction

The genus *Salmonella* is composed of two species, *Salmonella bongori* and *Salmonella enterica.* The former is a commensal of reptiles but causes gastroenteritis in humans. *S. enterica*, on the other hand, contains six subspecies. Remarkably, the *S. enterica* subspecies *enterica* is composed of nearly 3000 serovars, which cause diseases ranging from usually self-limiting gastroenteritis to potentially fatal typhoid fever. Fourteen million people per year are sickened by these bacteria [1]. *Salmonella* is a major public health problem, which is exacerbated by the lack of a licensed vaccine for invasive non-typhoidal *Salmonella* and the growing threat of multi-drug resistance. *Salmonella* remarkably secretes at least 49 type III effectors into host cells, which largely determines how this pathogen interacts with host cells.

Many of the virulence attributes of Gram-negative bacterial pathogens are attributable to type III secretion systems. These are highly complex bacterial structures that function as molecular syringes to inject effectors directly from bacterial cytosol into host cell cytosol, where they intimately engage the host in ways that promote pathogenesis. *S*. *bongori* possesses one type III secretion system, harbored by *Salmonella* pathogenicity island-1, whereas *S*. *enterica* additionally encodes *Salmonella* pathogenicity island-2. *Salmonella* pathogenicity island-1 and -2 were independently acquired. Each contains approximately 20 proteins that form the secretion apparatus, as well as effectors and chaperones. *Salmonella* utilizes *Salmonella* pathogenicity island-1 in the gastrointestinal stage of disease to invade the epithelium and to heighten the inflammatory response [2,3,4]. The host’s inflammatory response is intended to be protective but *Salmonella* exacerbates it to assist the establishment of infection. This reduces competition in the lumen of the gut, allowing *Salmonella* to surmount colonization resistance [5,6]. *Salmonella* pathogenicity island-2 effectors are secreted across the vacuolar membrane once the bacteria have been internalized within host cells, where they promote intracellular growth and permit the systemic dissemination of bacteria, among other things [7,8,9,10,11,12,13,14]. *Salmonella* was conventionally believed to only use *Salmonella* pathogenicity island-2 to facilitate intracellular survival and growth in the later stages of disease [7,8,9]; however, more recent studies demonstrated there is a fair amount of temporal and functional overlap between *Salmonella* pathogenicity island-1 and *Salmonella* pathogenicity island-2 [10,11,12,15]. In fact, *Salmonella* pathogenicity island-2 effectors are expressed in the gastrointestinal tract almost immediately and secreted prior to penetration of the epithelium [10,11,12,15]. Interestingly, most of the effectors secreted by *Salmonella* pathogenicity island-2 are located outside of it and were presumably acquired through independent horizontal acquisition events. They are all regulated by the *Salmonella* pathogenicity island-2 two-component regulator SsrAB, which may have interesting evolutionary implications [16].

### 1.1. Type III Effectors with Pro-Inflammatory Roles

The effects of the 49 known type III effectors of *Salmonella* are summarized in Table 1 and described below. The stimulation of inflammatory responses in the intestinal epithelium is a hallmark of disease caused by *S*. Typhimurium. *Salmonella* uses the type III secretion system encoded by *Salmonella* pathogenicity island-1 to inject multiple type III effectors into host cells that facilitate microbial invasion and are pro-inflammatory. Importantly, *Salmonella* next injects anti-inflammatory proteins into host cells to restore homeostasis. The anti-inflammatory effectors ensure that the host survives and short-circuits host mechanisms designed to prevent the dissemination of infected cells. SopE is injected into epithelial cells to reversibly activate the Rho-family GTPases Cdc42 and Rac1, whereas SopE2 only acts on Cdc42 [17,18]. When these two host proteins are GTP-bound, this drives actin polymerization, triggering actin-dependent ruffles and a macropinocytic event [19,20]. The *sopE2* gene is conserved amongst pathogenic strains of *Salmonella,* whereas SopE is encoded by a temperate bacteriophage not present in most strains [21]. SopB is an inositol polyphosphate phosphatase that stimulates Cdc42 and Rac [22]. In addition to SopE and SopE2, it contributes to actin cytoskeleton rearrangements and promotes invasion [22]. By injecting these effectors, *Salmonella* downregulates the host’s ability to dampen the inflammatory response. In addition to the Rho-family GTPases, downstream of the Toll-like receptors, these effectors activate a non-canonical signaling complex to drive inflammation [4,17,23,24]. Unrelated to invasion, SopB forms a signaling axis with Cdc42 and MEK1/2 to recruit vimentin around *Salmonella*-containing vacuoles, which forms a cage-like structure around them, preserving the bacterial vacuole [25]. This effector also activates Akt, deterring apoptosis [26].

SipA also promotes invasion. The C-terminal domain stabilizes F-actin and lowers the critical concentration of G-actin, inhibiting the depolymerization of actin filaments [27]. It is not required for entry but enhances the process [28]. It results in spatial localization and outward extension of membrane ruffles, facilitating efficient *Salmonella* uptake [27,29,30]. The N-terminal domain of SipA triggers the epithelium to synthesize and secrete apically the chemoattractant hepoxilin A3. It promotes the transmigration of polymorphonuclear neutrophils through the paracellular space to the luminal side of the barrier [31,32]. The two distinct functional motifs are interestingly physically separated by host caspace-3 cleavage [30].

*Salmonella* directly targets innate immune signaling mechanisms to stimulate inflammation with the type III effectors SopA and SopD. The induction of intestinal inflammation allows *Salmonella* to secure nutrients in the gastrointestinal tract. SopA targets TRIM56 and TRIM65, two host E3 ubiquitin ligases, which enhances interferon-ß expression through the RIG-1 and MDA5 innate immune receptors [33]. The type III effector SopD is required for pathogenesis [34]. SopD can stimulate inflammation with its GTPase-activating protein activity for Rab8, which normally negatively regulates inflammation [35].

SrfA, SrfB, and SrfC form a putative operon that is conserved amongst serovars of *Salmonella* associated with intestinal and systemic disease [16]. This apparent horizontal acquisition is 7 Kb in length and is flanked on each side by housekeeping genes [16]. The GC content of this putative operon is higher than the genome average (56% versus 52%), suggesting horizontal evolution [16]. SrfA was shown to be a novel *Salmonella* effector that promotes NFκB signaling to induce inflammation [36]. It is secreted into the cytoplasm during infection, where it interacts with the IRAK-1-Toll-interacting protein to disassociate it from IL-1R-associated kinase-1 (IRAK) [36]. The liberated IRAK-1 is phosphorylated and then activates the NFκB signaling pathway, which enhances the LPS-induced expression of pro-inflammatory cytokines [36]. A *srfA* deletion strain of *Salmonella pullorum* is much less virulent than the wild type in chickens and can potentially be used as a live attenuated vaccine [37]. SrfB and SrfC are predicted to be type III effectors but have no function ascribed to them [16].

The *sspH2* allele most commonly found in *S.* Typhimurium was reported to be pro-inflammatory [38]. A host-directed post-translational modification, palmitoylation, causes SspH2 to be incorporated into the host cell plasma membrane [39,40]. It was shown to interact with both NOD-1 and NOD-2 via the NF-κB pathway and enhances NOD-1-mediated IL-8 secretion [38,41]. Interestingly, SspH2 from *Salmonella enteritidis* C50336 was reported to be an anti-inflammatory effector whose deletion promoted the expression of IL-1β, IFN-γ, IL-12, and iNOS cytokines [42]. The different effects may be due to an allelic difference.

### 1.2. Type III Effectors That Promote the Systemic Spread of Infection

An emerging theme in host–pathogen interplay is a relatively new yet growing class of virulence genes that promote the systemic spread of pathogens. There has been considerable interest in studying how various *Salmonella* genes cause gastroenteritis and in understanding how some genes promote disease at systemic sites. However, relatively little has been invested in understanding how the bacteria transition from one phase of disease to the other. Gastrointestinal infections with *Salmonella* are usually self-limiting and rarely require hospitalization, whereas systemic infections are quite serious. Bloodstream infections are fatal up to 20% of the time, even with hospitalization [43]. In addition to their well-established roles in more traditional aspects of virulence, a growing number of both *Salmonella* pathogenicity island-1 and -2-associated genes have been implicated in influencing the adhesive and migratory properties of infected host cells. *Salmonella* pathogenicity island-2 structural mutants have been reported to have a 0–5-fold defect in intracellular growth over 24 h [7,8,9,44,45,46]. There are multiple rounds of cellular infection throughout the course of disease but it is still noteworthy that a mutant defective in *Salmonella* pathogenicity island-2 secretion is completely defective in colonizing the bloodstream of mice or chickens, regardless of the magnitude of the oral inoculum [46,47,48]. This suggests that the subversion of the adhesive and migratory properties of infected phagocytes by *Salmonella* may be a critical component of its virulence [10,11,12,49]. Perhaps the most logical phase of disease to interdict therapeutically is the transition from intestinal to systemic disease. Five type III effectors, SrfH/SseI, SpvC, SpvD, SopF, and SteC, were determined to play roles in the transition from one phase of disease to the other.

*Salmonella* spreads to the bloodstream from the gastrointestinal tract through a variety of different pathways. In the classical pathway, *Salmonella* invades and transits through the M cells of Peyer’s patches. The prevailing dogma in the field is that following consumption, *Salmonella* gains access to systemic tissue through the Peyer’s patches of the lymphatic system. The phagocytes that reside on the basal side of the M cells can then carry microbes to the regional lymph nodes. Secondary immune responses can be generated here but microbes that can survive within these phagocytes are thought to passively drain within them into the bloodstream. However, following oral ingestion, both *Salmonella* and *Yersinia* spread to internal organs of mice, which completely lack Peyer’s patches and in congenic control ones similarly [50,51]. Moreover, there is some evidence that the mesenteric lymph nodes contain oral infections to shield the host from systemic microbial dissemination, allowing the generation of a protective local immune response [50,52]. In one study, the authors showed that while increasing the number of dendritic cells in vivo with FLT-3L-induced expansion and simulating their migration with Toll-like receptor agonists resulted in more *S.* Typhimurium cells colonizing the mesenteric lymph nodes, there was no increase in the number that reached the blood. Additionally, they observed that when mice deficient in CCR7 were infected, almost no bacteria colonized the mesenteric lymph nodes; however, there was no defect in colonization of the spleen and liver [52].

More recently, a gut–vascular barrier was shown to control the translocation of antigens into the bloodstream while prohibiting the entry of the microbiota [53]. *Salmonella* was proposed to access the blood after increasing the permeability of the vasculature [53]. This may contribute to the extraintestinal dissemination of *Salmonella*, although its significance is hard to assess because of the observation that *Salmonella* pathogenicity island-1 mutants, which cannot efficiently penetrate the epithelium and invade endothelial cells, and thus are unable to efficiently access this pathway, or the lymphatic system for that matter, in multiple studies were reported to readily colonize the bloodstream [52,54].

In an alternative third route for enteropathogens to spread from the gut to the blood, the bacteria infect CX3CR1^+^ macrophages and dendritic cells that send dendrites through the gastrointestinal epithelium in the basal to apical direction to engage in antigen sampling. These cells can shuttle bacteria directly into the blood, in a manner that does not require *Salmonella* pathogenicity island-1 and does not disrupt the tight junctions of the intestinal epithelium or subsequently the vascular endothelium [10,11,12,54,55,56,57]. Non-pathogenic bacteria are not carried into the bloodstream [10]. *Salmonella* and likely other enteropathogens can induce these cells to disassociate from the basal face of the gastrointestinal epithelium and trigger them to then cross the blood vascular endothelium in the basal to apical direction. The tight junctions are not disrupted, as the phagocytes can express tight junction proteins themselves [10,11,12,54,55,56,57]. This process is termed reverse transmigration.

Additionally, *Salmonella* can invade the apical face of the epithelium; transit through the cells in a process that requires *Salmonella* pathogenicity island-2, although no individual effectors have been implicated; exit the basolateral face of the epithelium; and be taken up by macrophages in the lamina propria [58]. In this route, it was proposed that *Salmonella* was exploiting a constitutive antigen sampling pathway [58].

Finally, *Salmonella* can trigger necroptosis of epithelial cells with the *Salmonella* pathogenicity island-1 effector SopF and thereby gain access to the lamina propria [59]. Intestinal epithelial cells are one of the keys to host defense against systemic *Salmonella* dissemination. SopF is a *Salmonella* pathogenicity island-1 effector that maintains the integrity of the *Salmonella*-containing vacuole and allows the systemic spread of infection [59,60]. It was recently shown to affect systemic spread by modulating PANoptosis, which is a form of inflammatory-programmed cell death that has features of pyroptosis, necroptosis, and apoptosis [59]. PANoptosis helps the host control intestinal infections. A *sopF* mutant is significantly impaired versus wild-type bacteria for replication within macrophages and is significantly attenuated for virulence in mice [61]. SopF downregulates caspase-8 activation by activating phosphoinositide-dependent protein kinase-1 to phosphorylate p90 ribosomal S6 kinase [59]. Inactivated caspase-8 promotes necroptosis while inhibiting apoptosis and pyroptosis [59]. Apoptosis and pyroptosis limit *S.* Typhimurium replication through the expulsion of infected intestinal epithelial cells [62,63]. Biasing the host response in the intestinal epithelium toward necroptosis with SopF allows the bacteria to spread to deeper tissue.

The type III effector SrfH/SseI has at least seventeen naturally occurring alleles [64]. SrfH was intriguingly the first example of a virulence gene for which different alleles were shown to have opposite effects [10,11,65,66]. The dominant allele, SrfH Asp103, suppresses the productive motility of infected phagocytes, discouraging the systemic dissemination of infection, by deamidating the heterotrimeric G protein Gαi2 [13,67]. A strain expressing SrfH Asp103 is less likely than Δ*srfH* to colonize the mesenteric lymph nodes because the phagocytes infected with *S.* Typhimurium Asp103 do not migrate productively through the lymphatics [14]. Phagocytes infected with this strain are also unable to reverse transmigrate through the blood vascular endothelium into the bloodstream [11]. Interestingly, strains that encode SrfH Asp103 are almost always confined to the gastrointestinal tract of humans [64]. The naturally occurring SrfH Gly103 on the other hand stimulates the deadhesion or motility of infected phagocytes and encourages them to reverse transmigrate into the bloodstream [10,11,49]. Interestingly, the pseduolysogenization of SrfH Asp103 is one of the only differences between *S.* Typhimurium ST313 in Sub-Saharan Africa and in the UK and South America [68]. In the latter two locations, ST313 is almost always associated with gastroenteritis, whereas in Sub-Saharan Africa, it is highly invasive, even more so than *S.* Typhi [68,69]. Perhaps which allele of *srfH* a strain carries is one of the keys to determining if the bacteria cause gastrointestinal or systemic infection.

*Salmonella* Dublin does not cause gastroenteritis. It instead only produces systemic disease. Interestingly, the dominant *srfH* allele in serovar Dublin encodes a threonine reside at position 213 instead of a proline, which may affect how it promotes deadhesion or motility [64]. Thr213 is three residues away from His216, which is one of the three catalytic residues of the carboxyl terminus that deamidate the heterotrimeric G protein Gαi2, which produces non-polarized activation of it [67]. This results in either increased adhesion and/or the reduction of directed dendritic cell migration [13,67]. Perhaps SrfH Thr213 prevents this protein in *S.* Dublin from suppressing the deadhesion or productive motility of infected phagocytes, encouraging systemic dissemination.

Some *Salmonella* serovars harbor the virulence plasmid, which encodes a highly conserved locus called the *spv* (*Salmonella* plasmid virulence) operon [70]. These genes have long been thought to be important for human pathogenesis, as clinical isolates from patients with non-typhoidal bacteremia usually carry the *spv* operon [71,72]. SpvC is a phosphothreonine lyase that dephosphorylates Erk1/2, p38, and JNK [73,74]. A *spvC* mutant is not defective in replication within macrophages but was severely attenuated for virulence following oral infection in mice in one study and completely incapable of reaching the bloodstream in another [73,75].

By irreversibly dephosphorylating Erk1/2, p38, and JNK, SpvC dampens an inflammatory response and increases the motility of infected phagocytes. This allows the infected phagocytes to reverse transmigrate through the vascular endothelium in the basal to apical direction [12,65,66,76]. One of the pro-inflammatory cytokines is macrophage migration inhibitory factor (MIF). This cytokine has numerous functions but as the name suggests, was originally described as a soluble factor that potently inhibits the migration of phagocytes [77]. The secretion of SpvC may be how *Salmonella* overcomes this host mechanism intended to keep phagocytes in the area of infection, presumably to better fight it and to avoid inadvertently spreading it. SpvC is not required for bacterial survival within macrophages or the gastrointestinal tract; however, it is critical for systemic virulence [12,72,75]. Although it remains to be determined, it seems likely that many of the type III effectors that are pro-inflammatory will attenuate systemic dissemination, whereas those that are anti-inflammatory will enhance it. Overexpressing SpvC produces less TNFα, which upregulates MIF [73,78,79]. TNFα is upregulated by Erk1/2, p38, and JNK [80,81,82,83], which SpvC dephosphorylates [73,74]. In addition to regulating MIF, TNFα, among other things, promotes cell death [83,84]. Wild-type bacteria and Δ*spvC* survive identically within phagocytes, arguing against a role for cell death, which would have exposed the bacteria to gentamicin but does not exclude a role for MIF [73]. It will be important to assess the level of MIF that is produced and released by infected phagocytes and determine how this is influenced by SpvC and how this affects the migration of these cells.

In addition to SpvC, the *spv* operon of the virulence plasmid contains three other genes that may play roles in systemic spread. SpvB downregulates IKKβ to inhibit NF-κB activity [85]. It also depolymerizes actin and disrupts tight junctions [85]. SpvD uses its cysteine hydrolase activity to inhibit the nuclear transport of NF-κB p65 [85]. Interestingly, the amino acid sequence of SpvD is highly conserved amongst *Salmonella* serovars; however, residue 161, which is located close to the catalytic triad, in serovar Typhimurium, has an arginine, while the serovar Enteritidis possesses a glycine at this position [85]. SpvD Gly161 more potently inhibits NF-κB-mediated immune responses and this likely accounts for the increased systemic dissemination of *Salmonella* expressing this allele in mice [75,85]. It seems likely that many if not all of the anti-inflammatory type III effectors will enhance the systemic spread of infection.

SteC-mediated cytoskeleton manipulation is also required for *Salmonella* to induce infected phagocytes to reverse transmigrate [49]. SteC is the only *Salmonella* kinase effector [86,87]. *Salmonella* deploys SteC to induce the migration or invasion properties of macrophages by recruiting host myosin light chain protein Myl12a and phosphorylating Ser19 and Thr20 [49]. Interestingly, SteC can overcome ATP competition with host kinases by using four different NTPs as phosphate donors [49]. SteC causes actin rearrangement and enhances the migration and invasion of the bloodstream by infected macrophages [49].

Clarifying the role of the reverse transmigration pathway is necessary because of several published observations that suggest it plays an important, and until recently, largely overlooked role in the extraintestinal dissemination of enteropathogens. First, *Salmonella* pathogenicity island-1 mutants that cannot efficiently access the lymphatic system or the gut vascular barrier pathway still readily cause lethal infections. Second, both *Salmonella* and *Yersinia* colonize the hepatosplenic tissue of mice that lack Peyer’s patches, similarly to congenic control ones [50,51]. Further, Δ*spvC* is defective in triggering reverse transmigration in vitro and was shown to be incapable of colonizing the bloodstream of mice at an early time point, through the poorly characterized reverse transmigration pathway to the systemic circulation following oral infection [12]. The potential ramifications of these observations were fortified by the more recent finding in a different study that Δ*spvBCD* is completely unable to enter the blood and cause systemic disease in mice following oral infection at any time point even though it does not affect survival [12,73,75].

### 1.3. Modification of the Salmonella-Containing Vacuole by Type III Effectors

Many of the effectors secreted by *Salmonella* pathogenicity island-2 modify the vacuole the bacteria that reside within inside of host cells to make it more hospitable. The bacteria must be able to readily access nutrients and avoid the delivery of anti-bacterial factors to the *Salmonella*-containing vacuole.

SseF and SseG are two *Salmonella* pathogenicity island-2 effectors that are required for the *Salmonella*-containing vacuole in infected epithelial cells to localize near the Golgi network. They accomplish this by binding the Golgi resident protein ACBD3, counteracting the effectors SteA and PipB2 and microtubule motors that drive movement away from the Golgi [88,89,90]. They also inhibit autophagy in host cells by disrupting Rab1A signaling [91].

PipB and PipB2 are conserved amongst the various serovars of *Salmonella,* suggesting that they are necessary for virulence in different hosts. They localize to lipid rafts in the membranes of *Salmonella*-containing vacuoles and *Salmonella*-induced filaments [92]. PipB2, along with SifA, plays an essential role in the formation of tubules that emanate from the *Salmonella*-containing vacuole [93]. Interaction with PipB2 relieves the autoinhibition of the kinesin-1 molecular motor [93].

SifA is a well-studied effector whose prototypical function is stabilizing the *Salmonella*-containing vacuole [94]. It is also required for the formation of *Salmonella*-induced filaments (SIFs) in infected epithelial cells [95]. *Salmonella* residing within the *Salmonella*-containing vacuole or SIF continuum have higher metabolic activity than bacteria not associated with SIFs [96]. Thus, SifA allows *Salmonella* to overcome nutritional restriction in the vacuole by acquiring nutrients from the host cell endosomal system [96]. Additionally, SifA binds SKIP/PLEKHM2 to subvert the Rab9-dependent retrograde trafficking of the mannose-6-phosphate receptor, enhancing *Salmonella* growth by reducing lysosomal antibacterial activity [97]. SifA also exploits the host endolysosomal tethering factor HOPS complex to promote intravacuolar replication [98].

Interestingly, SifA is cleaved into two different functional domains by host caspase-3. Each domain functions independently. The N-terminal region binds SKIP, whereas the C-terminal region is a member of the WxxxE family of proteins, with possible GTPase mimicry function; however, no GEF activity has been observed in spite of interaction with the GTPases Rab7 and RhoA [99,100,101,102]. Caspase-3 cleavage is required for proper subcellular localization of SifA’s functional domains and is required for extraintestinal bacterial dissemination [99].

Separate from host cell motility, some SrfH localizes to peroxisomes, where it activates the host Ras GTPase, ADP-ribosylation factor-1 (ARF-1). ARF-1 activation recruits phosphatidylinositol-4,5-biphosphate-4 kinase and generates phosphatidylinositol-4,5-biphosphate on peroxisomes [103]. This facilitates the interaction of peroxisomes with lysosomes and the transfer of lysosomal cholesterol to *Salmonella*-containing vacuoles using peroxisomes as a bridge [103]. The cholesterol enhances *Salmonella*-containing vacuole stability and SIF integrity and facilitates bacterial growth [103].

SopD2 is closely related to SopD and is thought to have arisen through duplication and divergence [104]. The N-terminal domain of SopD2, which is unrelated to SopD, localizes the protein to endosomes and the *Salmonella*-containing vacuole [105]. SopD2 acts on Rab7, Rab8, Rab10, Rab32, and Rab34 [104,106,107]. SopD2 impairs the ability of Rab7 to bind the effectors RILP and FYCO1 with its N-terminus to prevent endosomes from delivering cargo to lysosomes [104].

SseJ is secreted by *Salmonella* pathogenicity island-2. It is present in intestinal serovars but is usually missing from strains from extraintestinal ones [108]. SseJ localizes to the cytoplasmic face of the *Salmonella*-containing vacuole. Along with SseL, it directs the eukaryotic lipid transporter oxysterol-binding protein 1 to the endosomal compartment in which *Salmonella* resides to promote vacuolar integrity [109]. SseJ has glycerophospholipid–cholesterol acyltransferase activity, which is activated by its interaction with GTP-RhoA [110,111,112]. It plays a role in the biogenesis of *Salmonella*-induced filaments and regulates the membrane dynamics of *Salmonella*-containing vacuoles [110,113,114].

The deletion of *sopD2* or *gtgE* severely attenuates *S.* Typhimurium proliferation within murine macrophages and reduces virulence in the mouse model of *S*. Typhimurium infection [106,115]. The bacteria target Rab32 with both SopD2 and GtgE to prevent the delivery of anti-microbial factors to the *Salmonella*-containing vacuole. Interestingly, when these two proteins are expressed in *S*. Typhi, which normally lacks them, they interfere with Rab32 recruitment and the bacteria are no longer restricted to growing in human macrophages but can survive within mouse macrophages as well [115].

SifB is always present in *Salmonella* serovars associated with intestinal disease but is usually non-functional in ones that cause extraintestinal illness. It shares a WxxxE motif with SifA and is recruited to the *Salmonella*-containing vacuole. It co-purifies with Rab10 and Rab13 but is not well-characterized [116].

### 1.4. Type III Effectors with Anti-Inflammatory Effects

The host’s inflammatory response elicited by a pathogen is intended to control infections by activating anti-microbial measures and regulating host cell death. Perhaps an overlooked component of the host response to infection is to limit the migration of infected cells. The host does not want to spread the infection, and by keeping phagocytes localized it can better fight it. While some alleles of *srfH*, *sopF*, spvC, *spvD*, and *steC* are known to promote the systemic spread of bacteria, it is likely that many of the anti-inflammatory effectors of *Salmonella* do so, and this will be important to test. There are numerous secreted *Salmonella* effectors with anti-inflammatory properties with dozens of homologs in other pathogens, many of which are required for serious disseminated disease [117,118,119]. A complete understanding of how these genes contribute to virulence could, thus, be useful in neutralizing the lethality of numerous intracellular pathogens.

SteE/SarA/GogC is secreted by *Salmonella* pathogenicity island-1 and -2 to initiate an anti-inflammatory transcriptional response. It is phosphorylated by the serine/threonine host kinase GSK3 and then remarkably reprograms it to phosphorylate the non-canonical signal transducer and activator of transcription 3 (STAT3) on a tyrosine residue [120,121]. Thus, SteE reprograms both the substrate and amino acid specificity of GSK3. The ensuant STAT3 activation along with GSK3 allows SteE to upregulate the anti-inflammatory M2 macrophage marker interlukin-4Rα [120,121]. Thus, by converting a host serine/threonine kinase into a tyrosine-directed kinase, SteE reprograms innate immunity by inducing the generation of an anti-inflammatory environment [120,121].

GogB is a type III effector encoded by the temperate bacteriophage Gifsy-1 in *S.* Typhimurium [122]. It can be secreted by both *Salmonella* pathogenicity island-1 and *Salmonella* pathogenicity island-2 but is translocated into host cells by *Salmonella* pathogenicity island-2. It targets host SCF E3-type ubiquitin ligase by interacting with the human F-box-only 22 protein and Skp1 [123]. In macrophages, GogB inhibits IκB degradation and limits NF-κB activation, dampening an inflammatory response [123]. Thus, GogB regulates inflammation-enhanced colonization by downregulating tissue damage.

AvrA plays an important role in attenuating host inflammatory responses. AvrA affects the mammalian intestinal immune response by inhibiting the MAPK JNK. AvrA reduces intestinal inflammation and the apoptosis of epithelial cells and reduces systemic cytokine responses [124,125]. AvrA deubiquinates IκBα, preventing its degradation, leading to the inhibition of NF-κB activation. It also blocks β-catenin degradation [126]. In *Salmonella* Enteritidis, AvrA suppresses autophagy by reducing Beclin-1 [127].

GtgA, PipA, and GogA are zinc metalloproteases that attenuate pro-inflammatory responses by cleaving the DNA binding loop of the NF-κB transcription factors p65 and RelB [128]. All *Salmonella* isolates encode at least one of these three virulence factors; thus, countering inflammation in this fashion is critical [128]. Interestingly, while a strain lacking these three effectors results in increased NF-κB stimulation, it is also more virulent [128]. Thus, the determinants attenuate virulence to preserve host homeostasis.

NF-κB signaling is also manipulated by SseK1, SseK2, and SseK3. These three effectors downregulate TNFα-stimulated NF-κB signaling [118,129]. This inhibition requires N-acetylglucosamine transfer to arginine residues, which requires a DXD motif in SeeK1 and SseK3. SseK proteins prevent TNFα-triggered cell death during macrophage infection [118].

SteA, SteB, and SteC were identified as being type III effectors when *S.* Typhimurium was mutagenized with a transposon that could generate translational fusions between arbitrary genes and the adenylate cyclase gene from *Bordetella pertussis* [86]. This adenylate cyclase generates cAMP only in the presence of calmodulin, which is found within eukaryotic cells but not bacteria. Macrophages were infected with pools of *S*. Typhimurium with arbitrary fusions to adenylate cyclase and screened using an ELISA for cAMP production in 96-well plates. The pools were then broken down, identifying SteA, SteB, and SteC [86]. SteA binds phosphatidylinositol 4-phosphate, which localizes it to the plasma membrane of infected host cells [130]. Mice infected with bacteria deleted for *steA* die before ones infected with wild-type *Salmonella*, suggesting that SteA suppresses immune responses [131]. SteA suppresses the degradation of IκB, which is the inhibitor of NF-κB [131]. SteA also controls the membrane dynamics of *Salmonella*-containing vacuoles. Cells infected with Δ*steA* bacteria versus wild-type display fewer SIFs, increased the clustering of *Salmonella*-containing vacuoles and abnormal vacuoles that contain more than one bacterium [132]. SteA is functionally linked to SseF and SseG and may influence microtubule motors on the bacterial vacuoles [132].

Intriguingly, SopD can manipulate the Rab8 GTPase to not only activate but also inhibit the inflammatory response [35]. SopD can activate Rab8 by displacing it from its cognate guanosine dissociation inhibitor [35]. This stimulates a signaling cascade that suppresses inflammation [35]. Thus, SopD is a bacterial effector that modulates the inflammatory response with both agonistic and antagonistic activity towards the same cellular target [35].

SspH1, along with SspH2 and SlrP, is a leucine-rich repeat protein with novel E3 ligase activity (NEL) activity. All three proteins are located outside of *Salmonella* pathogenicity island-1 and *Salmonella* pathogenicity island-2 [39]. The amino terminal leucine-rich repeat regions of these proteins confer specificity towards ubiquitination targets, whereas the carboxyl terminal region shows E3 ligase activity [41]. SspH1 and SspH2 can be secreted by *Salmonella* pathogenicity island-1 and *Salmonella* pathogenicity island-2 but are primarily secreted by the latter due to their expression patterns [133]. These three bacterial proteins are unrelated in sequence or structure to eukaryotic E3s [134]. SspH1 is anti-inflammatory. It localizes to the nucleus and targets the host protein PKN1, a serine/threonine protein kinase, to inhibit NFκB-dependent gene expression [135,136].

Remarkably, almost immediately following cellular ruffling and a macropinocytic event through which *Salmonella* enters host cells, the cells regain their normal architecture. The host cell recovery is mediated by the type III effector SptP. SptP is a GTPase-activating protein for Cdc42 and Rac, which turns these proteins off after they were stimulated initially by SopE/SopE2 and SopB [137]. The transient nature of ruffling is attributable to SptP having a longer half-life than SopE/SopE2 [138].

### 1.5. Type III Effectors That Cause the Death of Host Cells

Other than mediating attachment, when SipB is injected into macrophages, it associates with caspase-1 and induces apoptosis [139]. In addition to *Salmonella* pathogenicity island-1/SipB-mediated early apoptosis, *Salmonella* can also induce host cell apoptosis later in infection after injecting SseL with *Salmonella* pathogenicity island-2 [140,141]. SseL functions as a deubiquitinase enzyme in vivo. *Salmonella* infection induces the formation of ubiquitinated aggregates around the *Salmonella*-containing vacuole, which are targeted by the host cell’s autophagy machinery [142]. SseL counters this response by deubiquitinating the aggregates, which reduces autophagy in infected cells, enhancing *Salmonella* growth [142]. A *Salmonella sseL* mutant was defective for a delayed cytotoxic effect and is attenuated for virulence in a mouse model of typhoid fever [141]. SlrP is secreted by *Salmonella* pathogenicity island-1 and *Salmonella* pathogenicity island-2 [133,143]. It catalyzes the ubiquitination of mammalian thioredoxin, which leads to host cell death. It has also been reported to interfere with the folding ability of ERdj3, an endoplasmic reticulum chaperone, which can contribute to host cell death [144]. SlrP was also reported to ubiquitinate SNRPD2, a core component of the spliceosome [145].

### 1.6. Type III Effectors That Attenuate Virulence

CigR is a *Salmonella* pathogenicity island-2 effector, which is encoded by *Salmonella* pathogenicity island-3 [146]. It is an anti-virulence factor that intriguingly has functions inside both *Salmonella* and host cells [146,147]. Deleting *cigR* enhances survival and growth within human macrophages and renders the bacteria more lethal to mice [148,149]. CigR controls virulence by imposing a threshold for the virulence factor MgtC. When *Salmonella* first encounters MgtC-inducing conditions upon bacterial internalization within macrophages, the basal level of CigR is sufficient to bind MgtC and prevent it initiating its virulence program [150]. Inside of eukaryotic cells, CigR inhibits the development of the *Salmonella*-containing vacuole [147].

SseM is secreted by *Salmonella* pathogenicity island-2. It is found in many clinically relevant serovars and is found in all the subspecies of *Salmonella enterica.* It is co-transcribed with PipB2 [151]. It interacts with five components of the dystrophin-associated protein complex [151]. Interestingly, it is an anti-virulence gene, which attenuates the growth of *S.* Typhimurium within macrophages and modestly reduces its virulence in the mouse model of systemic disease [151].

### 1.7. Type III Effectors with Other Effects

The *sspH2* allele most commonly found in *S.* Typhimurium was reported to be pro-inflammatory. Intriguingly, a common *sspH2* allelic variant in *S.* Dublin contains a 60 bp in-frame deletion of a leucine-rich repeat [64]. The amino terminal leucine-rich repeat domains are believed to target the protein to its substrates. The in-frame deletion of one of the leucine-rich repeat domains seen in many *S.* Dublin strains may give the protein different sub-cellular targets. *S.* Dublin does not cause gastrointestinal illness where inflammation is critical but rather only causes systemic disease, which is promoted by the attenuation of inflammation. It will be interesting to determine what effect the *S.* Dublin allele that lacks one of the leucine-rich repeats has on inflammation and virulence. SspH2 from *S.* Typhimurium was originally described as being pro-inflammatory; however, a recent report found that it was primarily anti-inflammatory [152]. Cleary, more work is required to fully understand how the different alleles of *sspH2* affect virulence.

SrfJ is a *Salmonella* pathogenicity island-2 effector, whose amino acid sequence is 30% identical to human glucosylceramidase, which interestingly suggests that the gene was transferred horizontally from humans to *Salmonella* [16,153]. SrfJ was shown to act as a glucosylceramidase that modifies the lipdodome of epithelial cells in a way that alters the transcription of genes involved in cell communication, the immune response, and cell death [154].

SipB, SipC, and SipD play roles in the formation of the *Salmonella* pathogenicity island-1 needle complex. They are necessary for the bacteria to attach to mammalian cells [155,156,157,158,159,160]. SipC promotes bacterial entry into host cells by bundling F-actin [158,161]. SipD resides on the tip of the needle, where it regulates the secretion of proteins [160]. Bacteria lacking SipB, SipC, or SipD are deficient in intimate attachment to host cells [160].

SteD is a *Salmonella* pathogenicity island-2 effector that ubiquitinates and reduces surface-localized mature MHC class II molecules (mMHCII). SteD localizes to the Golgi network and to vesicles that contain the E3 ubiquitin ligase MARCH8 and mMHCII [162]. SteD uses MARCH8 to ubiquitinate and surface-deplete mMHCII to suppress T cell activation during *Salmonella* infections both in vitro and in mice [162].

SteB is one of the few known *Salmonella* type III effectors that remains uncharacterized. It presumably plays a role in intestinal disease, as it is found in serovars of *Salmonella* associated with gastroenteritis but is usually non-functional in those that cause extraintestinal infections [108].

## 2. Discussion

A fair number of the type III effectors of *Salmonella* have either homologs or functional analogs in related organisms. Learning more about how they function in *Salmonella* pathogenesis may enhance our understanding of the virulence of related microorganisms. They thus may help establish a general underlying framework through which pathogens manipulate host cells. While a great deal of progress has been made over the years in ascribing molecular functions to these effectors in *Salmonella*, much work remains. Many of the type III effectors of *Salmonella* either induce or attenuate inflammation. The pathogen goes to considerable lengths to exacerbate inflammation to establish infection but then deploys numerous anti-inflammatory effectors to dampen the host response. The former is necessary to establish infection in the gastrointestinal tract, whereas the latter is required to return the host to homeostasis and to potentially allow the infection to proceed to the bloodstream. It has been apparent for some time that most effectors have multiple functions and that some may act in concert with each other. Additionally, we are just beginning to appreciate that different alleles of virulence genes can have different, and in some cases opposed, effects on host cells. Most of the work described here was performed with *S.* Typhimurium. This organism causes systemic infection in mice but typically produces relatively mild, self-limiting gastroenteritis in humans. It will be important to explore in the future what effect allelic variation amongst the type III effectors has on the pathogenesis of serovars and strains that are more problematic for people.

Comparative genomics can tell us much about type III effectors in *Salmonella*. Conserved amongst both typhoidal and non-typhoidal serovars is a core group including SteA, SteD, SseL, SseG, SptP, SpiC, SopD, SopB, SipD, SipC, SipA, SipB, SifA, PipA, PipB, and PipB2 [117]. This group of effectors likely play fundamental roles in *Salmonella* pathogenesis that are common amongst the various serovars. While virulence genes such as these are typically noted as being present or absent, it is worth noting that in the case of SrfH and SpvD, different naturally occurring alleles of the same virulence gene were shown to change the course of infection [11,163]. Following up on these findings, a bioinformatics study found that dominant alleles of ten genes, including *srfH*/*sseI*, *sadA*, *siiE*, *bapA*, *sseK2*, *sopA*, *sifB*, *shdA*, *sopE,* and *stfH*, were found in strains that cause intestinal disease, whereas dominant alleles of ten additional genes, *ratB*, *sseL*, *sspH2*, *fliC*, *zirC*, *steC*, *steA*, *slrP*, *mgtB*, and *fimH,* were identified only in strains associated with invasive disease [64]. The impact on the virulence of nearly all allelic variation amongst *Salmonella* serovars and strains remains to be determined. Something that has received scant attention is the possibility of epistatic interactions amongst the various type III effector alleles.

An additional layer of complexity is the fact that some virulence genes have overlapping functions and are believed to act in concert with each other. There is not a clear consensus on whether the type III effectors of *Salmonella* act together or individually affect virulence. In one study, the secretion of only five *Salmonella* pathogenicity island-2 effectors, SifA, SseF, SseG, SopD2, and SteA, was necessary to promote survival and replication within macrophages [75]. This report indicated that in addition to these five genes, the *spv* operon was necessary to cause systemic disease in the murine model of typhoid fever [75]. This is consistent with the observation that SpvC is necessary for *Salmonella* to colonize the bloodstream of mice following oral inoculation [12]. However, in a different study, 48 effectors (both *Salmonella* pathogenicity island-1 and *Salmonella* pathogenicity island-2 ones) were tested for persistence in mice [149]. Each of the 48 effectors was individually replaced with a specific DNA barcode and mice were co-infected with wild-type bacteria and a mutant and a competitive index was determined [149]. Individually mutating all but seven of the 48 effectors reduced the persistence of infection, indicating that most of the effectors are required for virulence, and this argues against redundant functions [149]. One of the challenges for the field going forward is to determine under which circumstances individual effectors contribute to virulence.

The pro-inflammatory type III effectors of *S*. Typhimurium have been well-studied, and the ones with anti-inflammatory properties have been characterized for their abilities to promote survival within host cells and to abrogate apoptosis [117,128,164]. There are, however, any number of effectors such as SpvC and SpvD with anti-inflammatory properties that could encourage the migration of infected host cells in addition to promoting survival within them or by inhibiting pro-apoptotic signaling. It is interesting that the *spv* operon has a variable distribution in non-typhoidal *Salmonella* but is conserved amongst clinical isolates from human blood [11,12,71,72]. SpvC and SpvD are not just required for rapid bacteremia but for it at all, despite the observation that they do not mediate survival within the gastrointestinal tract or within host cells [10,12,75]. This result suggests that SpvC and SpvD are required not just for traveling through the reverse transmigration pathway but also for disseminating through the lymphatic system [75]. It will, thus, be important to test whether SpvC and SpvD are necessary to allow the movement of infected phagocytes through both lymphatic vessels and endothelial monolayers, with its phosphothreonine lyase activity against the MAPK kinases. By irreversibly dephosphorylating the MAPK kinases, SpvC dampens an inflammatory response, facilitating the systemic spread of infection. It seems likely that suppression of the host cytokine MIF is essential to allowing the infection to disseminate beyond the gastrointestinal tract, and this will be important to test. Perhaps MIF acts as a host checkpoint, balancing the need to ultimately resolve inflammatory responses with inhibiting the spread of microbes. The neutralization of this checkpoint may be a shared strategy amongst intracellular pathogens, which could one day be therapeutically modulated. Many diverse pathogens of humans that spread from their initial site of colonization to systemic tissue to cause more serious disease express SpvC homologs [74]. Although it remains to be tested, it seems likely that most if not all pro-inflammatory effectors will deter the systemic spread of intracellular bacteria. Conversely, presumably many if not all of the effectors that are anti-inflammatory will promote the intra-host dissemination of internalized bacteria. In fact, one of the major functions of effectors that attenuate host inflammation may be to encourage the movement of infected host cells.

It is noteworthy that inhibiting the host’s inflammatory response prevents *Salmonella* from replicating in the gastrointestinal tract but enhances microbial dissemination to systemic tissue. Thus, countering microbial factors that enhance inflammation might produce more serious disease. There are, however, numerous effectors with anti-inflammatory properties that may promote virulence not just by countering the host’s conventional anti-microbial mechanisms but also by allowing the movement of infected phagocytes. Perhaps these virulence factors could serve as a new class of anti-microbial targets.

## Figures and Tables

**Table 1:** *Salmonella* type III effectors.

Effector	SPI-1/SPI-2	Binding Partner (s)	Function
Sop	+/−	Cdc42, Rac1, and Rab	Rho GTPase exchange factor, promotes bacterial invasion by triggering membrane ruffling.
SopE2	+/−	Cdc42 and Rac	Similar to SopE.
SopB	+/−	Cdc42	Inositol phosphatase required for chloride secretion and neutrophil recruitment. Interdicts inositol phosphatase singling pathways and induces Akt activation.
SipA	+/−	Caspase-3, F-actin, T-plastin, syntaxin8	Regulates concentration, polymerization, and stability of actin molecules at the site of bacterial entry.
SopA	+/−	TRIM56, TRIM65, UbcH5a, UbcH5c, UbcH7, HsRMA1, Caspase-3	Induces fluid secretion and the inflammatory response. Involved in inflammation and inducing PMN migration.
SopD	+/+		GAP and a GEF for Rab8.
SrfA	+/−	IRAK-1-Toll interacting protein	Activates NFκB signaling. Disassociates IRAK-1-Toll interacting protein from IL-1R-associated kinase-1.
SrfB	ND		Putative type III effector.
SrfC	ND		Putative type III effector.
SspH2	−/+	UbcH5-ubiquitin, SGT1, NOD1	Activates NOD1 signaling.
SopF	+/−	*ATP6V0C*, *ARF1*	Attenuates intestinal epithelial cell inflammation, allowing systemic dissemination, among other things.
SrfH (SseI) Asp103	−/+	IQGAP-1, Gαi2	Inhibits directional migration of phagocytes.
SrfH (SseI) Gly103	−/+	TRIP6, IQGAP-1, Gαi2	Promotes deadhesion/motility of phagocytes.
SpvC	+/+	Erk1/2, p38, JNK	Inhibits MAPK signaling.
SpvB	ND/+	G-actin	Inhibits F-actin polymerization, promotes macrophage apoptosis.
SpvD	+/+	PKN1, Ube2D XPO2	Inhibits NF-κB signaling.
SseF	ND/+	SseG, ACBD3	Tethers SCV to Golgi.
SseG	ND/+	SseF, ACBD3	Tethers SCV to Golgi.
PipB	ND/+	PDZD8	
PipB2	+/+	Kinesin-1, KIF5B, annexin A2	Recruits kinesin-1 to SCV to reorganize late endosome/lysosome to facilitate bacterial survival.
SifA	ND/+	*PLEKHM1*, *PLEKHM2*, *GDP-RhoA*, *Rab7*, *Caspase-3*	Induces SIF formation, detoxifies lysosomes, maintains vacuolar membrane stability.
SopD2	ND/+	Rab7, Rab32^+^	Promotes bacterial replication within host cells. Contributes to *Salmonella*-induced filament formation.
SseJ	−/+	GTP-RhoA, cholesterol	Esterification of cholesterol.
SifB	ND/+	Co-purifies with Rab10 and Rab13	ND
GtgE	+/+	Rab32, Rab29, Rab38	Prevents Rabs from accumulating on SCVs.
SifB	−/+	Co-purifies with Rab10 and Rab13	Poorly characterized.
SteE	+/+	GSK3*α/β*, *STAT3*	Transcriptional reprogramming to anti-inflammatory phenotype.
GogB	+/+	SKP1, FBXO22	Inhibits NF-κB signaling.
AvrA	+/+	ERK2, MK4, MK7, p53	Inhibits inflammation (NF-κB signaling) and apoptosis.
GtgA	+/+	Class II NF-κBs	Inhibits NF-κB signaling.
PipA	−/+	NF-κB p65	hibits NF-κB signaling.
GogA	+/+	P65, RelB	Inhibits NF-κB signaling.
SseK1	+/+	FADD, TRADD	Inhibits TNFα-stimulated NF-κB signaling and necroptosis.
SseK2	ND/+	ND	Inhibits TNFα-stimulated NF-κB signaling and necroptosis.
SseK3	ND/+	TRIM32, TRADD	Inhibits TNFα-stimulated NF-κB signaling and necroptosis.
SteA	+/+	PI (4) P	SIT formation.
SteB	+/+	ND	ND
SteC	−/+	MEK1, HSP27, Myl12a	Stimulates the assembly of F-actin network around SCV, enhances migration and invasion of macrophages through the vascular endothelium.
SspH1	+/+	PKN1	E3 ubiquitin ligase. Inhibits androgen steroid receptor signaling.
SlrP	+/+	ERdj3, TRX1	E3 ubiquitin ligase. Inhibits IL-1β release. Attenuates inflammation.
SptP	+/−	Cdc42, Rac1, VCP, vimentin, cSrc, NSF, Syk	Downregulates membrane ruffling, ERK and MAPK activation, and secretion of proinflammatory cytokines.
SipB			Formation of the SPI-1 T3SS needle complex, caspase-1-induced apoptosis, and release of IL-18.
SseL	ND/+	OSBP, Ubiquitin	Induces late macrophage death
CigR			Anti-virulence effectors. Inhibits SCV development and replication.
SseM	−/+	dystrophin-associated protein complex	Attenuates intracellular growth and virulence.
SrfJ	+/+	glucosylceramidase	Affects cell communication, the immune response and cell death.
SipC	+/−	Cytokeratin 8, cytokeratin 18, Exo70, F-actin, syntaxin 6	Formation of the SPI-1 T3SS needle complex, targets F-actin to allow invasion.
SipD	+/−		Formation of the SPI-1 T3SS needle complex, promotes the secretion of effectors.
SteD	−/+	MHCII, MARCH8	Inhibits antigen presentation.
SseB	−/+		Part of the SPI-2 translocon.
SseC	−/+		Part of the SPI-2 translocon.
SseD	−/+		Part of the SPI-2 translocon.

## Data Availability

No new data were created or analyzed in this study.
